# Intraoperative pain management for patients undergoing medication-assisted rehabilitation: a scoping review

**DOI:** 10.1186/s12871-025-03538-5

**Published:** 2025-12-10

**Authors:** Marte Henriksen, Sidsel Ellingsen, Hege Kristin Aslaksen Kaldheim

**Affiliations:** 1 Department of Health and Nursing Science, University of Agder, Jon Lilletuns vei 9, Grimstad, 4879 Norway; 2 Oslo University Hospital, Ullevål, Oslo, Norway; 3 Department of Nursing, VID Specialized University Ulriksdal 10, Bergen, 5007 Norway

**Keywords:** Intraoperative, Medication treatment of opioid use disorder, Nurse anaesthetist, Anaesthesiologist, Analgesia, Buprenorphine, Methadone, Naltrexone

## Abstract

**Introduction:**

Managing pain relief during the intraoperative phase for patients receiving medication-assisted treatment for opioid use disorder is both challenging and complex. This patient group can have or develop increased opioid tolerance and heightened pain sensitivity. Both require a different approach to pain management compared with patients not receiving such treatment. This scoping review aimed to identify and map the literature on intraoperative pain management for patients receiving medication for opioid use disorder.

**Methods:**

A systematic search of Embase (OVID), Medline and CINAHL, was undertaken in December 2023. All types of literature published from 2013 to 2023 were included, except for abstracts. A follow-up search was conducted in April 2025.

**Results:**

Eight articles were included: one qualitative study, one case study report, one expert Delphi study and five non-research articles. The results suggest a paucity of research on intraoperative pain management for patients undergoing medications for opioid use disorder.

**Conclusion:**

Limited evidence exists regarding intraoperative pain management for patients undergoing medication-assisted treatment for opioid addiction. Despite this gap, existing literature discusses the roles of methadone, buprenorphine and naltrexone in the context of surgical treatment. Multimodal strategies can effectively manage pain for this complex group, and collaboration between nurse anaesthetists and anaesthesiologists is crucial for optimal pain management and patient safety.

**Supplementary Information:**

The online version contains supplementary material available at 10.1186/s12871-025-03538-5.

## Introduction

 A person with opioid addiction has developed a physical and/or psychological dependence on opioids, which include prescription analgesics, such as morphine, and illegal drugs, such as heroin [1]. Worldwide, opioid misuse and opioid use disorders have become increasingly common [2, 3]. Each surgical patient has the right to be treated with dignity, which includes high-quality pain assessment and management [4]. Managing acute pain among people with opioid use disorder (OUD) presents challenges because they need a larger opioid dose to gain an analgesic effect. A patient with OUD is at risk for opioid-related side effects, such as respiratory depression, constipation and addiction [5].

Research has found that almost one fifth to one third of surgical patients have opioid tolerance [6], which entails substantial problems with effective pain management that include heightened pain sensitivity and, because of their opioid tolerance, a reduced effectiveness of opioid medications. Opioid withdrawal can lead to physiologic stress responses [7], and this group of patients is often underestimated and undertreated, as many healthcare providers report a knowledge gap and lack of confidence in managing acute pain for people with OUD [8, 9].

Patients receiving medication for opioid use disorder (MOUD) are a complex group of surgical patients with regard to pain management. MOUD is a specialized multidisciplinary treatment for people with OUD receiving substitution treatment with medications such as methadone, buprenorphine and naltrexone as a part of a comprehensive rehabilitation programme [8]. Methadone is an effective medication for managing opioid withdrawal symptoms [8]. Buprenorphine is often administered as a sublingual tablet in combination with naloxone to avoid opioid withdrawal. The benefit of buprenorphine is that it is more accessible and more effective for withdrawal/craving prevention compared to full-agonist opioids, such as methadone [8]. It is effective at treating opioid dependence while attenuating the effects of other opioids, making it a safer drug than methadone, due to decreased risk of drug interactions. Buprenorphine is also more cost-effective than methadone [8]. Naltrexone is an opioid antagonist that exists in both short- and long-acting formulations. Due to its mechanism of action, it causes withdrawal in people who are taking full-agonist opioids [8]. MOUD offers an effective approach to overcoming addiction and supporting individuals on their journey to recovery [8].

Managing pain relief under surgery for the patients who are using MOUD is both challenging and complex, requiring extensive knowledge and experience [10]. This patient group may be anxious about receiving adequate pain relief due to the stigma associated with opioid use and being judged by the surgical staff [8]. The patients who are using MOUD, needs to feel confident that they can trust the surgical team through clear and effective communication. Additionally, patients on MOUD and the surgical team must have a mutual understanding that the patient’s pain management will be fully respected [8]. Therefore, a multidisciplinary collaboration between the anaesthesiologists, nurse anaesthetist, surgeons and perioperative nurses is essential [11]. Further, the surgical team should have advanced team skills, such as communication and decision-making, as well as interpersonal skills. This complex patient group needs a different approach regarding pain management compared with patients not on such treatment [12], as patients who are using MOUD have increased opioid tolerance [6, 13, 14] and may have heightened pain sensitivity [12]. In addition, this patient group reports higher pain scores, longer resolution of pain [6], and longer hospital stays compared with opioid-naïve patients [7]. It is essential that the nurse anaesthetist promote person-centred care, by showing respect for the patient as a unique human being and ensure that the patient is involved in their own care by planning the intraoperative setting [15].

The treatment of pain consists mostly of the administration of opioids. However, as scientific knowledge has emerged regarding pain mechanisms, the development of other types of medications to address pain has advanced. Multimodal pain management combines analgesics with different modes or sites of action [16].

Information and evidence-based knowledge regarding pain management for patients who are using MOUD in the intraoperative phase seem scarce. Thus, this scoping review aimed to identify and map the literature on intraoperative pain management for patients receiving medication for OUD. The research questions were as follows:

1. How does the literature describe the use of methadone, buprenorphine and naltrexone in the intraoperative phase and pain management for medication for opioid use disorder?
2. How does the literature describe the use of multimodal pain management for patients receiving medication for opioid use disorder?
3. How does the literature describe the collaboration between nurse anaesthetists and anaesthesiologists in the intraoperative pain management process?

## Methods

### Design

A scoping review was performed to identify and map the literature related to the study’s aim. Scoping reviews are useful to identify literature irrespective of study design and to discern key concepts, undercover knowledge gaps and determine the need for further research [17, 18]. This scoping review used the Joanna Briggs Institute framework [17], which includes the following steps: (1) identifying the research question(s); (2) identifying relevant literature; (3) selecting appropriate literature; (4) charting data; and [5] collating, summarising and reporting the results. The results are reported following the Preferred Reporting Items for Systematic Reviews and Meta-Analyses extension for Scoping Reviews (PRISMA-ScR) guidelines (Appendix, 3) [19]. The review protocol was not preregistered, and ethical approval was not required.

### Inclusion criteria

The population, concept and context framework were used to identify the concepts studied and develop the eligibility criteria [17]. Our target population was patients receiving MOUD. The concept to explore was pain management during the intraoperative phase. The context was care for surgical patients receiving MOUD in the intraoperative phase.

### Target population

The target population was female and male patients over 18 years of age receiving MOUD with an American Society of Anaesthesiologist Physical Status Classification System (ASA) 1–5. The ASA physical status classification system offers a simple categorization of a patient’s physiological status to help predict operative risk [20]. Pregnant patients, patients with chronic pain and patients receiving palliative care were excluded (see Table 1 for the inclusion criteria protocol).

**Table 1:** Inclusion criteria protocol

	Inclusion	Exclusion
Participants	Patients receiving medications for opioid use disorder (MOUD)	Patients under 18 years old Pregnant patients Patients with chronic pain Patients receiving palliative care
Concepts	Pain management during the intraoperative period.	Surgical treatment that does not include general anesthesia
Context	Care for surgical patients (under MOUD) in the intraoperative period	Preoperative and postoperative period
Types of evidence sources	Qualitative, quantitative, reviews and literature	Abstract
Year	2013–2025	Literature before 2013
Language	Danish, Englisch, Norwegian and Swedish	Literature in other language

### Concept to explore

The concept to explore was intraoperative pain management for patients undergoing surgery that involved general anaesthesia. General anaesthesia is designed to achieve unconsciousness (hypnosis) and immobilization during surgical procedures [21, 22]. Multimodal pain relief during general anaesthesia was included. This is defined as the use of more than one modality of pain control to achieve effective analgesia while reducing opioid-related side effects (16, 23).

### Context

The context was intraoperative anaesthesia care in operating rooms, trauma rooms and surgical units. The context also included nurse anaesthetists in the surgical team.

### Identifying relevant literature

The first and last authors, together with a research librarian, devised the search strategy at the beginning of September 2023. An initial search was performed to identify pertinent search terms. The search terms were then decided through consultations among the research team. The first author and the research librarian undertook a systematic search in Embase (OVID), Medline and CINAHL in December 2023. Boolean operators were used to combine the following keywords related to substance-related disorders: (addict* OR ((opioid* OR opiate* OR morphine* OR heroin* OR opium* OR substance* OR drug*) adj2 (disorder* OR misus* OR use* OR using* OR addict* OR abuse* OR dependen*))).ti, ab. On anaesthesia, the following keywords were searched: (sedat* OR anesthe* OR anaesthe* OR ((anesthe* OR anaesthe*) adj1 (opioidfree OR opioidfree OR “opiate-free” OR “opiate free”))).ti, ab. The following keywords on intraoperative care were also searched: ((perioperative OR peri-operative OR intraoperative OR intra-operativ OR intraoperative OR surg* OR perianesthe*) adj2 (care OR nurse anesthetists *)).ti, ab. All types of literature published from 2013 to 2023 in Danish, English, Norwegian or Swedish were included, except for publications consisting only of abstracts. The research team also searched the reference lists of the selected studies to identify any additional articles. The last author and the research librarian conducted a follow-up search in April 2025 to check if additional literature had been published since the original search. The full search terms, strategy and results for each database are shown in Appendixes 1 and 2.

### Selecting appropriate literature

Initially, 687 articles were identified (CINAHL: *n* = 253; Embase: *n* = 330; Medline: *n* = 104). The results were imported into EndNote 21, where 123 duplicates were removed. The EndNote library of 564 articles was then exported into Rayyan, a free web-based screening tool [24]. The first and last authors developed a screening manual based on the inclusion and exclusion criteria (see Table 1 for the inclusion criteria protocol). Then, the first and last authors independently screened the titles and abstracts using the blind mode function in Rayyan, following the scoping review methodology. A total of 18 articles were included for full-text reading and were read by the first and last authors. These two authors discussed the full-text article’s relevance to the aim and whether they met the inclusion criteria. When disagreements arose, the article was read and discussed in more depth to determine its significance and inform the decision. Finally, seven articles that met the inclusion criteria were included in this scoping review. Searching the reference lists did not add any more articles. The follow-up search in April 2025 identified 102 articles (CINAHL: *n* = 24; Embase: *n* = 65; Medline: *n* = 13). The results were imported into EndNote, where one duplicate was removed. A total of 101 articles were read according to their titles and abstracts. Five articles were selected for full-text reading, and one article was included.

### Quality appraisal

Scoping reviews do not include quality appraisals, but such appraisals can demonstrate transparency in the review process. In this scoping review, we used the Critical Appraisal Skill Programme (CASP) checklist (2025) for one qualitative article [25] and the Delphi Critical Appraisal Tool for one expert Delphi study (26). Additionally, we used the Joanna Briggs Institute checklist for the case reports in one article [27]. Finally, we evaluated five articles [28–34] according to the approach in the article by Hek and Langton [33], on critically appraising ‘non-research’ literature [34].

### Charting data

The eight included articles were read several times, and a patterning chart was used to assess the key themes in the included articles [35]. The themes arose from an inductive, thematic analysis in which the scoping review’s aim, and research questions were used as the starting point. The charting data also included the title, author, publication year, country of origin and journal, study design/method, participants and sample size [34, 35] (see Table 2). All the authors in the research group discussed the data charting and themes.

**Table 2:** Key themes and patterning chart

Themes and articles	The use and consideration of medication methadone, buprenorphine and naltrexone during the intraoperative phase	Multimodal pain management and regional anaesthesia used during the intraoperative phase	Collaboration among healthcare professionals	Attitude and demeanor of the nurse anaesthetists and anaesthesiologist
Barreveld et al. (2023) [28]	X	X	X	
Coluzzi et al. (2017) [29]	X	X	X	X
Forsberg et al. (2018) [25]			X	X
Goyal et al. (2013) [27]	X	X	X	
Kohan et al. (2021) [26]	X	X	X	X
Kohan et al. (2025) [30]	X	X	X	X
Torp & McClain. (2020) [32]	X	X	X	
Oesterle et al. (2019) [31]	X	X		

## Results

**Fig. 1:**
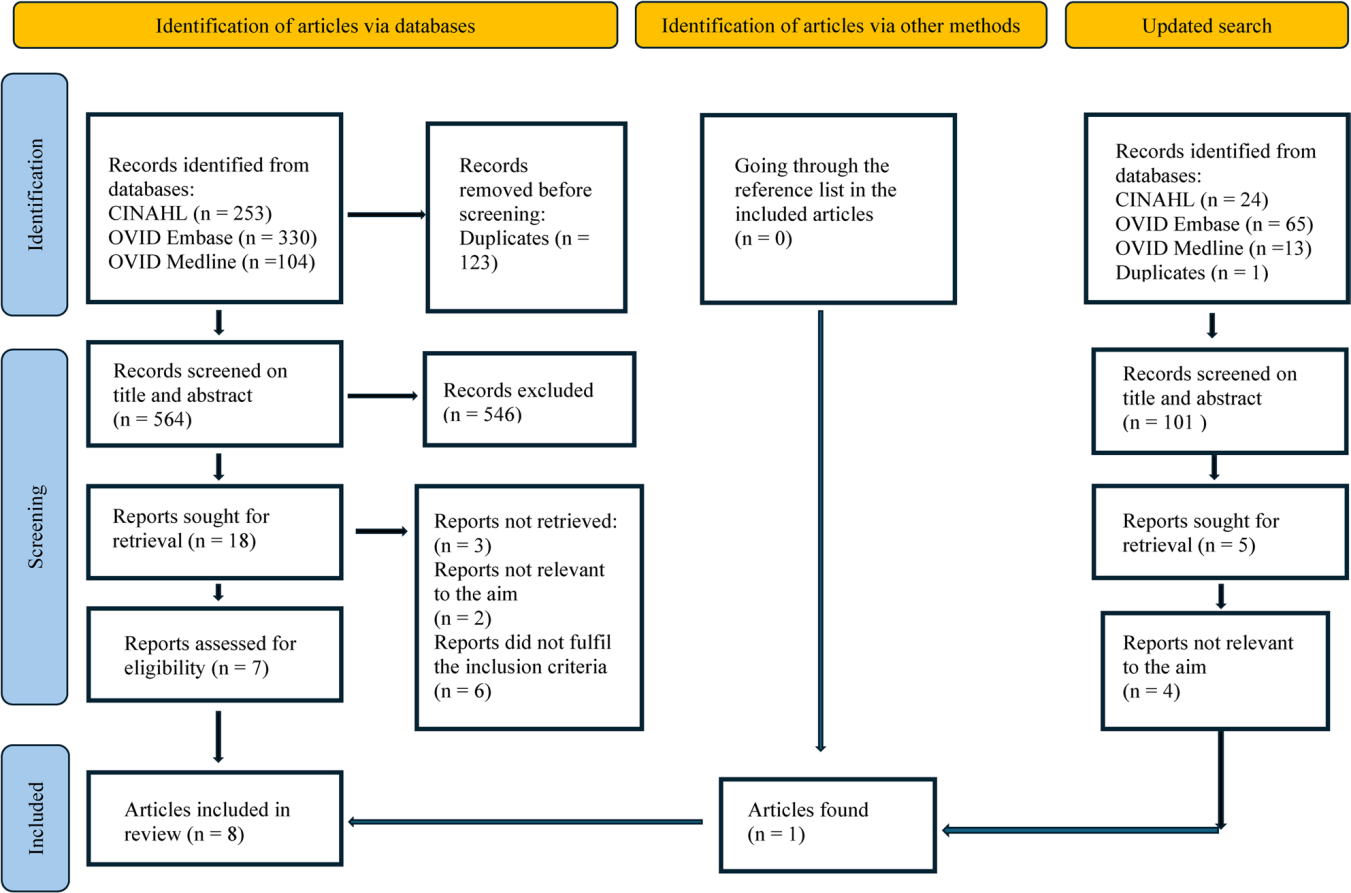
PRISMA-ScR flow diagram

This scoping review included eight articles (see Fig. 1 PRISMA-ScR flow diagram).

The eight articles comprised one qualitative study [25], one case study report [27], one expert Delphi study (26) and five non-research articles [28–34]. The low number may reflect a lack of research.

### Key characteristics

The included articles’ characteristics are summarized in Table 3. Of the eight articles included, one was published in 2025, one in 2023, one in 2021, one in 2020, one in 2019, one in 2018, one in 2017 and one in 2013. The articles originated from four dissimilar countries: India, Italy, Sweden and the United States of America.

**Table 3:** Description of the included articles

Author(s), year, country	Title	Type of article	Aim/theme	Design/methods/ participants	Key findings	Conclusion
Barreveld et al. 2023, USA [28]	Caring for our patients with opioid use disorder in the perioperative period: A guide for the anesthesiologist	Non-research article	This article underscores the role of interdisciplinary collaboration in the perioperative phase for patients undergoing MAT treatment. It explores the physiological and psychological effects of MAT treatment and their implications for anesthesia planning. Patients with MAT treatment face an increased risk of aspiration, cardiovascular events, and other complications, making a team-based approach crucial. The article also reviews the medications methadone, buprenorphine, and naltrexone, which play a significant role in managing pain in this context.	Non	Non	Anesthesia personnel play a crucial role during the perioperative phase. A systematic, step-by-step approach to patient care, the use of multimodal pain relief, and effective teamwork all contribute to improved patient outcomes. In conclusion, there is a need for more research in this area, particularly studies focusing on patients undergoing LAR treatment who are also undergoing surgery.
Coluzzi et al. 2017, Italy [29]	The challenge of perioperative pain management in opioid-tolerant patients	Non-research article	The article focuses on patients undergoing MAT treatment in the perioperative phase. It stresses the importance of distinguishing between abusers, former abusers, and those in pharmacological treatment. Additionally, it provides a thorough review of the management of patients under maintenance dose with methadone, buprenorphine, and naltrexone. This comprehensive review aims to equip healthcare professionals with the knowledge and confidence to handle such cases effectively. The article also addresses attitudes and common misconceptions about MAT treatment.	Non	Non	The article concludes that the number of opioid-tolerant patients is increasing and that managing them in the perioperative phase can be challenging. The article also provides ten pieces of advice, mentioning both effective pain relief and multimodal pain relief.
Forsberg et al. 2018, Sweden [25]	Nurse anesthetists’ reflection on caring for patients with previous substance dependence: Balancing between professionalism and preconceptions	Research article	The study aim was to describe nurse anesthetists’ reflections on the provision of perioperative care to patients with previous substance dependence.	A qualitative approach with a descriptive design. Semistructured interviews based on clinical vignettes were conducted with 10 nurse anesthetists.	The perioperative care provided to patients with previous substance dependence was perceived as balancing between professionalism and preconceptions for this specific patient group. The nurse anesthetists felt that anesthetizing this group of patients constituted a challenge with regard to knowledge, experience, and time. However, the nurses also had feelings of distrust and uncertainty because of lack of knowledge.	The nurse anesthetists strove to uphold the principle that patients who are/have been substance dependent have the same right to adequate treatment and care as all patients. If guidelines were developed for this patient group, care could be made safer and nurses’ sense of uncertainty minimized.
Goyal et al. 2013, India [27]	Anesthesia for opioid addict: Challenges for perioperative physician	Case rapport article	The case study involves a pharmacy employee who has been grappling with opioid dependence for a significant 7-year period. The patient used naltrexone for the maintenance of opioid dependence.	Case rapport. One patient aged 35-year-old opioid dependence	Non-opioid anesthesia was chosen (naltrexone was continued, as well as ketamine, sevoflurane, diclofenac and paracetamol). The patient had an increased need for pain relief postoperatively.	The case report concludes that anesthesia personnel play a key role in maintaining the patient’s opioid requirements, administering supplemental opioids in the perioperative and postoperative periods, and in addition providing non-opioid medications and nerve blocks when needed.
Kohan et al. 2021, USA [26]	Buprenorphine management in the perioperative period: educational review and recommendations from a multisociety expert panel	Research article	The purpose of this multisociety collaborative document, based on literature review and expert opinion, is to serve as an educational resource for physicians focused on recognizing and managing opioid use disorder in the perioperative period. Specifically, the document will provide information on buprenorphine pharmacology, the perioperative management of patients on buprenorphine for opioid use disorder, and the advantages of initiation of buprenorphine postoperatively in patients with suspected opioid use disorder.	Delphi process was used to assess the literature and expert opinion for each topic, with 100% consensus being achieved on the statements and each recommendation.	The expert opinion addresses MAT treatment, focusing mainly on buprenorphine in the perioperative phase. The systematic literature review also addresses the lack of time and the desire for more research.	Experts have concluded that increasing the use of buprenorphine during the perioperative phase is a vital strategy to reduce both morbidity and mortality. The purpose of this expert statement is to encourage anesthesia professionals to employ evidence-based treatment options for patients on long-acting reversible (MAT) medications and to tailor treatments to individual patient needs.
Kohan et al. 2025, USA [30]	Narrative review: Managing buprenorphine and opioid use disorder in the perioperative setting	Non-research article	Summarise the American Society of Anesthesiology and Pain Medicine Multisociety Working Group Practice Advisory recommendations on existing opioid misuse and/or opioid use disorder (OUD). treatment barriers and perioperative management best practices. Also to demonstrate the benefits that greater involvement of the anesthesiologist can have in managing patients with OUD perioperatively	Non	Barries to treatment include stigma, inadequate education and training, delivery system fragmentation, regulatory and legal barriers, insurance coverage, and inadequate reimbursement that disincentivizes care. Stigma also exists towards the use of Federal Drug Agency-approved medications for the treatment of OUD (MOUD)	We are faced with the perioperative consequences of the opioid crisis for many years to come. Expansion to access to treatment is greatly needed. All clinicians, regardless of specialty, will be required to receive education in OUD prior to renewing their DEA license in efforts to increase clinicians’ ability to recognize, refer, and treat OUD. Anesthesiolgists, who have a knowledge base in analgesia, can provide these services in the perioperative arena. Doing so may provide life-saving treatment.
Oesterle et al. 2019, USA [31]	Medication-assisted treatment for opioid use disorder	Non-research article	The article offers specific guidance on using MAT medications in the perioperative phase.	Non	Non	The article concludes that the opioid crisis presents an opportunity to change our perspective on pain management. We need to look beyond simply using drugs for pain relief. Additionally, it is crucial to develop a better framework for addressing this issue in the future.
Torp et al. 2020, USA [32]	Perioperativ pain control in the opioid dependent patient: just bite the bullet	Non-research article	The article emphasizes the necessity of a comprehensive approach to pain management, particularly focusing on reducing the reliance on opioids. It also underscores the significance of multimodal pain management and discusses the specific use of drugs such as buprenorphine, methadone, and naltrexone. Furthermore, the article highlights the role of regional anesthesia in managing pain effectively.	Non	Non	The article concludes that patients who are dependent on opioids present a challenge to the healthcare system. It emphasizes the importance of effective communication within the surgical team and suggests that multimodal pain relief methods and regional anesthesia are the most effective strategies for reducing or potentially eliminating the need for opioids altogether.

Forsberg et al.’s [25] qualitative descriptive study aimed to describe nurse anaesthetists’ reflections on the provision of intraoperative care to patients with previous substance dependence. Semi-structured interviews with 10 nurse anaesthetists were performed [25]. Goyal et al. [27] presented the case of a 35-year-old pharmacy worker with a 7-year history of opioid dependency who was undergoing treatment with MOUD. The authors discussed the challenges faced in the intraoperative phase to provide effective pain relief without resorting to opioids. In the Delphi study, a multisociety working group on OUD representing pain medicine, addiction and pharmacy health science developed guidance for anaesthesiologists and pain physicians on managing patients with current opioid disorder in the intraoperative phase [26].

The articles by Barreveld et al. [28] and Coluzzi et al. [29] included patients undergoing MOUD and how this treatment affects anaesthesia planning and intraoperative care. Oesterle et al. [31] provided recommendations for the use of opioid substitution therapy medication during the intraoperative phase. Torp and McClain [32] highlighted the need for a holistic approach to pain relief with a particular emphasis on minimizing opioid use. Kohan et al.’s [30] addressed existing opioid misuse and/or OUD treatment barriers and perioperative management best practices.

### Key themes of the included articles

Four key themes emerged: (1) the use and consideration of methadone, buprenorphine and naltrexone medication during the intraoperative phase; (2) multimodal pain management and regional anaesthesia during the intraoperative phase; (3) collaboration among healthcare professionals; and (4) attitude and demeanour of the nurse anaesthetist and anaesthesiologist (Table 2). 

#### Use and consideration of methadone, buprenorphine and naltrexone medication during the intraoperative phase

All the included articles, except for the article by Forsberg et al. [25], addressed the MOUD medications methadone, buprenorphine and naltrexone. Barreveld et al. [28] claimed that buprenorphine is best continued throughout the perioperative period. Coluzzi et al. [29] noted that no data favour one specific opioid over another for surgical patients with opioid dependence and emphasize the unpredictability of intraoperative tolerance levels. Barreveld et al. [28] recommended that patients who are using MOUD continue on daily methadone doses and that the use of non-opioid medications and regional anaesthesia is maximized. In acute surgery, if the daily dose has not been received, an equivalent dose should be administered, and oral methadone can be converted to intravenous methadone by administering one third to one half of the daily dose over 24 h. Coluzzi et al. [29], Oesterle et al. [31] and Torp and McClain [32] supported this recommendation. Methadone’s conversion between dosage forms is inconsistent, but it can also be administered intramuscularly or subcutaneously if oral intake is not possible. Intravenous methadone is more established for intraoperative use [29].

Buprenorphine should continue through the intraoperative phase, with patients taking their regular daily dose on the day of surgery (26, 28–31). If opioids are necessary, opioids with high intrinsic activity, such as intravenous fentanyl should be administered [28, 29]. Higher opioid doses may be necessary to compete with buprenorphine [29]. Tapering off buprenorphine can cause withdrawal symptoms and a relapse, so the daily dose should continue with concomitant high intrinsic activity opioids for optimal pain management [32]. Kohan et al. [26, 30] supported the combination of buprenorphine with opioids such as fentanyl for effective pain management and emphasized the importance of individual dosing based on expected pain levels and available regional anaesthesia.

Patients using naltrexone represent a challenging group, and research their management is limited. Long-term naltrexone users who discontinue the medication before surgery may have reduced opioid sensitivity, requiring low initial opioid doses titrated to the desired effect [28, 29]. Non-opioid medications and regional anaesthesia should be maximized [28]. Oesterle et al. [31] and Torp and McClain [32] suggested stopping naltrexone before surgery, with oral naltrexone being discontinued 72 hours prior. Goyal et al. [27] reported a case in which the patient continued naltrexone on the day of surgery, opting for non-opioid-based anaesthesia. Barreveld et al. [28] claim that naltrexone dosed for OUD may be discontinued before elective surgeries where acute opioids are anticipated to be needed” [28]. If opioid-based anaesthesia had been planned, naltrexone would have been discontinued 24–72 h before surgery [27, 29]. If naltrexone was stopped, the time interval to restarting naltrexone should be considered on a case-by-case basis coordinated with the outpatient prescriber and surgical team [29].

#### Multimodal pain management and regional anaesthesia

Seven of the eight articles included the key theme of multimodal pain management and agreed that a combination of different pain management methods, including multimodal analgesia and regional anaesthesia, can be effective, especially for patients receiving MOUD [26– 32]. Barreveld et al. [28] explained that a pain regimen consisting of non-opioid medications and non-pharmacological methods can help reduce postoperative pain, limit opioid requirements and decrease opioid-related side effects throughout the intraoperative phase. Barreveld et al. [28] and Torp and McClain [32] provided an overview of non-opioid medications that can be administered during the intraoperative phase, namely, ketamine, lidocaine, acetaminophen, non-steroidal anti-inflammatory drugs, gabapentin, magnesium and dexmedetomidine.

Ketamine is described as an effective analgesic for patients undergoing MOUD in the intraoperative phase [29–32]. It is known to reduce opioid consumption and improve the quality of pain management in cases of chronic opioid exposure [28, 32].

Intravenous lidocaine infusion is also mentioned as advantageous when regional anaesthesia is unavailable, and an infusion of 2.0 mg/kg preoperatively has proven to be as effective as epidural infusion [32]. Furthermore, dexmedetomidine has gained popularity throughout the intraoperative phase and has been shown to reduce opioid consumption in patients undergoing MOUD [32].

Regional anaesthesia is a beneficial choice for intraoperative pain relief in patients undergoing MOUD (26–30, 32) because it has an opioid-sparing effect [29–32], reduces complications associated with opioids, improves safety, achieves better pain control, contributes to less stress, prevents opioid-included hyperalgesia and allows for lower doses of general anaesthesia, thereby having a positive effect on multiple organ systems [32].

#### Collaboration among healthcare professionals

Seven articles encompassed the key theme of collaboration among healthcare professionals [25–32] and stating that collaboration between different professionals is important. Three articles addressed the anaesthesiologist as the leader throughout the intraoperative phase, who playing an essential role in guiding and ensuring effective pain management [26– 28]. Barreveld et al. [28] highlighted that anaesthesiologists must recognize the existing gap in patient care for patients who are using MOUD and be aware that stigma surrounding this patient group can lead to less effective pain treatment during surgical procedures. Kohan et al. [26, 30] stated that a lack of this theme in healthcare professionals’ education is also a barrier and suggested that the use of evidence-based knowledge and a greater focus on this patient group can increase the understanding and willingness to provide good care for this patient group.

Coluzzi et al. [29] and Torp and McClain [32] emphasized the importance of the surgical team and healthcare professionals being knowledgeable about intraoperative pain management for patients who are using MOUD. Forsberg et al. [25] stated that in the intraoperative phase, close collaboration between the anaesthesiologists and the nurse anaesthetist, is vital. It is also crucial because the nurse anaesthetist is often responsible for the patient’s anaesthesia management, either under the direct or indirect supervision of the anaesthesiologist. Kohan et al. [30] noted that a lack of confidence among health professionals generally regarding opioid misuse and/or OUD, MOUD and perioperative management best practices.

#### Attitude and demeanour of the nurse anaesthetists and anaesthesiologist

Five of the eight articles discuss the attitude and demeanour of the nurse anaesthetists and anaesthesiologist [25–32]. Patients undergoing MOUD can be misjudged and unfairly treated; some fear receiving inadequate pain relief, and many experience a sense of shame (26). Forsberg et al.’s [25] study focused on the attitudes of nurse anaesthetists towards this patient group. Based on what the nurse anaesthetists had read in the patients’ medical records, several of them felt that this patient group was prejudged before they entered the operating room [25]. Some nurse anaesthetists prejudged patients with a history of drug addiction and have negative attitudes because these patients can be perceived as immoral, with character defects and little likelihood of recovery from their addiction [25, 29]. Kohan et al. [30] also noted that stigma exists towards the use of MOUD.

Time constraints and how they can impact the pain management of patients who are using MOUD were also mentioned. The nurse anaesthetists in Forsberg et al.’s [25] study, suggested that a plan should be established during the preoperative conversation with the patient, but a lack of time was found to contribute to why this was not done [25]. Barreveld et al. [28] stated that if a patient undergoing MOUD is scheduled for surgery, anaesthesiologists have a golden opportunity to establish an individual pain management plan for the patient. However, this requires anaesthesiologists to take or find the time to do so.

## Discussion

The primary aim was to identify and map the literature on intraoperative pain management for patients receiving medication for OUD. The results suggest a paucity of research. Given that patients who are using MOUD are a very vulnerable and complicated group of surgical patients, further research is needed. Pain relief is a human right, and all patients should have access to pain management without discrimination [36]. Five articles [28–34] were non-research articles, while three articles were in line with scientific requirements according to the design and method described [25–29]. This lack of research was noted by Macintyre et al. [6] and Kohan et al. (26, 30). Bordi [37], observed that there is scarce evidence supporting the optimally management of pain in patients undergoing MOUD in the intraoperative phase. Although there is a paucity of data on patients who are using MOUD considerations in the intraoperative setting, there were some acknowledgements in the literature and expert consensus for perioperative pain management that can be transferable and used for the intraoperative period [6, 38– 39].

In the included articles, there was a consensus that patients who are using MOUD using methadone, should continue with their daily dose throughout the intraoperative phase (26, 28–32). Methadone is widely used to help patients experiencing opioid addiction, and it contributes to reducing and stabilizing both cravings and withdrawal symptoms [40]. The nurse anaesthetist and anaesthesiologist must be observant of unwanted side effects and use their clinical judgement in the intraoperative phase because this patient group requires more opioids (37). Buprenorphine prescribed as a treatment for opioid dependence to patients who are using MOUD makes this patient group more demanding and complex because there are more factors to consider. One guideline recommended discontinuing naltrexone therapy before surgery but acknowledges uncertainty about the management of methadone and buprenorphine [38]. Recent data suggest that discontinuation of buprenorphine before surgery is not required [41]. Evidence regarding acute pain management in traumatically injured patients undergoing outpatient buprenorphine therapy states that continuing this therapy in acute trauma patients decreases daily opioids compared to patients who discontinue buprenorphine [41]. This finding may be transferable to intraoperative pain management practices and aligns with this scoping review’s recommendation that buprenorphine be continued through the intraoperative phase, with patients taking their regular daily dose on the day of surgery (26, 28–31). This was also supported by Lembke et al. [39] in their editorial, which highlighted that buprenorphine should continue through the perioperative period [39]. Interest in MOUD continuation perioperatively has increased [26, 39, 42]. Li et al. [43] they reported that perioperative patients who continued preoperative buprenorphine received fewer postoperative opioids than patients whose buprenorphine was held or tapered [43]. Goyal et al. [27] described a case in which naltrexone was administered throughout the surgical procedure, but the patient received a non-opioid form of anaesthesia. Naltrexone should be discontinued between 24 and 72 h prior to surgery if the patient is to be given an opioid-based form of anaesthesia [29–31– 32].

Pain incorporates a sensory and emotional experiences, and managing pain is a multidimensional approach that requires individualization. Including the patient as an active partner can improve pain management [44]. Good communication and provided information seem essential [12]. If the patients who are using MOUD is to be prepared and tapered off buprenorphine before surgery, person-centred pain management needs to be implemented [15]. Person-centred care can improve healthcare quality, patient safety and outcomes [45– 47] and nurse anaesthetists and anaesthesiologists should promote person-centred care, including showing respect for the patient as a unique human being and ensuring that the patient is involved in their own care in the intraoperative setting [15]. Inadequate pain management can have serious consequences for the patient and the hospital, such as adverse events and prolonged care [48].

Pain perception is a subjective experience that differs among individuals at the cerebral level [49]. Predicting a patient’s who is using MOUD tolerance to pain seems impossible because pain is complex [12]. It seems central to involve the patients using MOUD and to adopt a person-centred approach because such care is grounded in each patient’s experiences and needs [50]. This was supported in Kohan et al.’s [30] article, which stated that care should be tailored to the individual patient’s needs and should consider the type of MOUD the patient is receiving. The patients who are using MOUD perspective, earlier experiences and health records, together with the nurse anaesthetists’ and anaesthesiologists’ competence, seem essential and might prevent misconceptions about pain management during the intraoperative phase [29, 51]. One misconception is that maintenance treatment with methadone or buprenorphine provides pain relief [29]. While methadone and buprenorphine are both opioid medications, their analgesic effects are shorter (4–8 h) than the duration of the patient’s opioid withdrawal symptoms (24–48 h). Patients who are opioid-tolerant require more opioids. Furthermore, this group experiences increased sensitivity to pain, feeling pain more intensely and with pain subsiding more slowly [29]. Another misconception is that additional opioids for pain relief can cause a relapse. This is not evidence-based, and it appears that potential stress and undertreated pain can be risk factors for relapse [29]. In the article by Quaye et al. [52], the authors aimed to determine whether there is a difference in pain scores and opioid consumption after elective surgery in patients receiving MOUD. They found that patients who switched from buprenorphine to methadone experienced the most significant increase in postoperative opioid consumption. However, they noted that more evidence is needed to support this finding [52]. Given the scarcity of information on intraoperative pain treatment for patients who are using MOUD, there is a significant need for further research in this area. Consequently, there will be assumptions regarding this patient group. In one study on postoperative pain after elective surgery on patients who are using MOUD, the authors found significant differences in opioid utilization between patients maintained on buprenorphine and those maintained on methadone, as well as increases when the patient’s respective MOUD medications were not administered within 72 h postoperatively. Additionally, they found that buprenorphine and methadone did not interfere with analgesic outcomes in the postoperative phase and that buprenorphine, when continued into the postoperative phase, improved analgesic outcomes [52], as supported by Lembke et al. [39].

Multimodal analgesia uses more than one modality of pain control to achieve effective analgesia while reducing opioid-related side effects [23]. It can result in better pain control and fewer side effects through opioid sparing [16]. Our results show that multimodal pain management may be an effective approach for managing pain in patients who are using MOUD in the pre-, intra- and postoperative phases [26– 32]. This result is weak since there is scarce evidence in the literature about intraoperative pain management for patients who are using MOUD. Evidence exists on multimodal analgesia used in the postoperative phase generally for surgical patients, although these studies often remain insufficient, and patients have reported unrelieved pain during their postoperative hospitalization [53– 55]. Khali et al. [54] highlighted the importance of personalized, multimodal pain management approaches to improve postoperative care and enhance recovery. This may support our results that a combination of different pain management methods, including multimodal analgesia, for example, combining maximization of non-opioids and regional anaesthesia, can contribute to achieving optimal pain relief for surgical patients undergoing MOUD [26– 32]. It seems that this complex patient group needs a different approach to pain management compared with patients not receiving MOUD treatment [12].

Non-pharmacological multimodal approaches to pain management and sedation that can be utilized in the intraoperative setting include music medicine. Research suggests that music medicine can reduce the need for opioid and sedative medications [56, 57]. It may be beneficial for patients on MOUD and warrants further investigation.

The multidisciplinary surgical team includes anaesthesiologists, nurse anaesthetists, surgeons and perioperative nurses, who collaborate and depend on each other to deliver high-quality care for surgical patients [10, 58]. Good teamwork is based on optimal cooperation between the different professions and characterized by respect and understanding for each other’s tasks [59– 60]. Managing intraoperative pain requires a multidisciplinary approach and especially close collaboration between nurse anaesthetists and anaesthesiologists. This is extremely important for patients who are using MOUD during the intraoperative phase [10]. The results of this study emphasize the importance of collaboration between nurse anaesthetists and anaesthesiologists to ensure optimal pain management and patient safety [25– 26, 28– 30, 32].

The nurse anaesthetist has responsibility for the patient’s anaesthetic treatment with direct or indirect supervision by the anaesthesiologist [25]. Therefore, it seems essential that the anaesthesiologist accepts the nurse anaesthetist’s way of working [60]. Nurse anaesthetists aim to deliver holistic nursing care based on the philosophy of person-centred care [46], and this seems vital when there is evidence that the nurse anaesthetist’s behaviour can affect the patient’s pain experiences [51]. When the nurse anaesthetist acts professionally and in a caring way with the patient, it is beneficial [51]. The nurse anaesthetists in the study by Forsberg et al. [25] opined that planning and implementing anaesthesia for patients who are using MOUD can be very challenging. According to the nurse anaesthetists’ assessments, it requires expertise, experience and time, as well as an open mind and a developed intuition, all of which take many years to develop [25]. Knowledge and experience and a positive attitude are crucial for providing quality anaesthesia care to patients who are using MOUD. It appears that a negative attitude is associated with a lack of knowledge, which may affect the quality of care of patients who are using MOUD [61]. This is supported by the results of Forsberg et al. [25], who found negative attitudes among the nurse anaesthetists in their study, who said that they experienced distrust and uncertainty around patients who are using MOUD because of their lack of knowledge. The nurse anaesthetists felt that they had deficient knowledge, which resulted in insecurity. It is vital that nurse anaesthetists ask all patients, including those with addiction issues, relevant questions and discuss the approach to be taken with those receiving substitution treatment with addictive drugs. This is because addictive and anaesthetic drugs interfere with each other [62].

Kristoffersen et al. [58] wrote about the preanaesthetic assessment clinic as an opportunity for the patient and healthcare professional to exchange information. The goal is to ensure that surgical patients are thoroughly prepared for surgery. Nurse anaesthetists working in a preanaesthetic assessment clinic experienced this as a place for developing knowledge and perceived an increase in their competence [58]. For patients who are using MOUD, a preanaesthetic assessment clinic could be a unique opportunity to meet nurse anaesthetists who have specific knowledge regarding their situation and how to best manage intraoperative pain. This could prevent patients who are using MOUD from experiencing prejudice and negative attitudes that result in feelings of shame and fear. Furthermore, it could hinder misconceptions and the undertreatment of pain [25].

## Strengths and limitations

This scoping review’s strength is that the methodological quality assessment used the Joanna Briggs Institute framework, involving all the authors. One limitation is that published material such as grey literature and abstracts was excluded, and data may have been missed. Another limitation is the search strategy used to select the literature. It should have included the perioperative phase in addition to the intraoperative phase, as this omission excluded important and relevant studies. The literature concerning perioperative pain management could have been beneficial for this patient population. To overcome this limitation, some of this literature is included in the discussion. A further limitation is the quality of the included literature, particularly the non-research articles. This resulted in limited evidence and insights into the topic.

## Conclusion

This scoping review offers insights into the literature on intraoperative pain management for patients who are using MOUD and shows that there are significant gaps in the evidence about this field. The included literature makes it problematic to provide a generalizable conclusion, but the articles include descriptions of the use of methadone, buprenorphine and naltrexone for the intraoperative phase and pain management. Additionally, the articles show that multimodal pain management can be effective for surgical patients undergoing MOUD. The included literature also describes the importance of close collaboration between nurse anaesthetists and anaesthesiologists in the intraoperative pain management process to ensure optimal pain management and patient safety. The development of knowledge and experience among healthcare providers in this field is also observed to be crucial for providing quality anaesthesia care to patients who are using MOUD. A preanaesthetic assessment clinic may prevent patients who are using MOUD from experiencing prejudice and negative attitudes that lead to feelings of shame and fear. Furthermore, it could prevent misconceptions and the undertreatment of pain.

## Supplementary Information

Supplementary Material 1: Appendix 1. Search historic 2023.

Supplementary Material 1: Appendix 2. Updated search 2025.

Supplementary Material 1: Appendix 3. PRISMA-ScR check list.

## Data Availability

No datasets were generated or analysed during the current study.

## Abbreviations

ASA: American Society of Anaesthesiologists Physical Status Classification System
CASP: Critical Appraisal Skill Programme
MOUD: Medication for Opioid Use Disorder
OUD: Opioid use disorder
