# Supplementary material for: Intraoperative pain management for patients undergoing medication-assisted rehabilitation: a scoping review
Source: BMC Anesthesiol. 2025 Dec 10;26:40. doi: 10.1186/s12871-025-03538-5 (PMC12801853; doi:10.1186/s12871-025-03538-5)
Supplement: Supplementary file 1 — Supplementary Material 1: Appendix 1. Search historic 2023. [file 12871_2025_3538_MOESM1_ESM.pdf]

APPENDIX 1 Search historic

Database: Ovid MEDLINE(R) and Epub Ahead of Print, In-Process, In-Data-Review & Other Non-Indexed Citations and Daily <1946 to December 07, 2023> Search Strategy:

**1**	exp Opioid-Related Disorders/ (34505)
**2**	Substance-Related Disorders/ (106139)
**3**	(addict* or ((opioid* or opiate* or morphine* or heroin* or opium* or substance* or drug*) adj2 (disorder* or misus* or use* or using or addict* or abuse* or dependen*))).ti,ab. (357171)
**4**	Opiate Substitution Treatment/ (4734)
**5**	((buprenorphine* or methadone* or naltrexone* or opiate* or opioid*) adj3 ("maintenan* therap*" or "maintenan* program*" or "maintenance treatment*" or "replacement therap*" or "substitution treatment*" or "substitution therap*")).ti,ab. (5385)
**6**	1 or 2 or 3 or 4 or 5 (401612)
**7**	Pain Management/ (41214)
**8**	Analgesics, Opioid/ (60628)
**9**	exp Analgesics/ (591584)
**10**	(analges* or ((pain or analges*) adj2 (management or relie*))).ti,ab. (206164)
**11**	Anesthesia/ (66804)
**12**	(sedat* or anesthe* or anaesthe* or ((anesthe* or anaesthe*) adj1 (opioid-free or "opioid free" or opiate-free or "opiate free"))).ti,ab. (470669)
**13**	7 or 8 or 9 or 10 or 11 or 12 (1136139)
**14**	Perioperative Care/ (16098)
**15**	Perioperative Nursing/ (6984)
**16**	Intraoperative Care/ (17692)
**17**	((perioperative or peri-operative or intraoperative or intra-operative or peroperative or surg* or perianesthe*) adj2 (care or nurs*)).ti,ab. (31692)
**18**	14 or 15 or 16 or 17 (67876)
**19**	6 and 13 and 18 (659)
**20**	limit 19 to (yr="2013 -Current" and (danish or english or norwegian or swedish)) (439)
**21**	Nurse Anesthetists/ (2923)
**22**	Nurses/ (46416)
**23**	Anesthetists/ (486)
**24**	Anesthesiologists/ (1931)
**25**	(nurs* or anesthetist* or anesthesiologist* or (nurs* adj1 an?esthe*)).ti,ab. (556393)
**26**	21 or 22 or 23 or 24 or 25 (570649)
**27**	6 and 13 and 18 and 26 (164)
**28**	limit 27 to (yr="2013 -Current" and (danish or english or norwegian or swedish)) (104)

Database: **Embase** <1974 to 2023 December 07>
Search Strategy:

**1**	opiate addiction/ (29512)
**2**	drug dependence/ (69652)
**3**	(addict* or ((opioid* or opiate* or morphine* or heroin* or opium* or substance* or drug*) adj2 (disorder* or misus* or use* or using or addict* or abuse* or dependen*))).ti,ab. (496586)
**4**	opiate substitution treatment/ (3230)
**5**	((buprenorphine* or methadone* or naltrexone* or opiate* or opioid*) adj3 ("maintenan* therap*" or "maintenan* program*" or "maintenance treatment*" or "replacement therap*" or "substitution treatment*" or "substitution therap*")).ti,ab. (7365)
**6**	1 or 2 or 3 or 4 or 5 (526172)
**7**	analgesia/ (154666)
**8**	analgesic agent/ (105029)
**9**	anesthesia/ (114328)
**10**	(analges* or ((pain or analges*) adj2 (management or relie*))).ti,ab. (293940)
**11**	(sedat* or anesthe* or anaesthe* or ((anesthe* or anaesthe*) adj1 (opioid-free or "opioid free" or opiate-free or "opiate free"))).ti,ab. (619632)
**12**	7 or 8 or 9 or 10 or 11 (950498)
**13**	perioperative care/ or peroperative care/ (17120)
**14**	perioperative period/ or intraoperative period/ (114147)
**15**	perioperative nursing/ or operating room nursing/ or perianesthesia nursing/ (6248)
**16**	peroperative care/ (15181)
**17**	((perioperative or peri-operative or per-operative or peri-surgical or perisurgical or intraoperative or intra-operative or peroperative or surg* or perianesthe*) adj2 (care or nurs* or period*)).ti,ab. (73160)
**18**	13 or 14 or 15 or 16 or 17 (189052)
**19**	6 and 12 and 18 (2109)
**20**	limit 19 to ((danish or english or norwegian or swedish) and yr="2018 - Current") (1123)
**21**	nurse anesthetist/ (2622)
**22**	Nurses/ (122933)
**23**	anesthesist/ (27774)
**24**	anesthesiologist/ (12278)
**25**	(nurs* or anesthetist* or anesthesiologist* or ((nurs* or staff or personnel) adj1 an?esthe*)).ti,ab. (654178)
**26**	21 or 22 or 23 or 24 or 25 (698911)
**27**	6 and 12 and 18 and 26 (471)
**28**	limit 27 to ((danish or english or norwegian or swedish) and yr="2013 - Current") (330)

Database CINAHL

S29	S7 AND S14 AND S21 AND S27	Limiters - Published Date: 20130101-20231207; Language: Danish, English, Norwegian, Swedish Search modes - Boolean/Phrase	253
S28	S7 AND S14 AND S21 AND S27	Expanders - Apply equivalent subjects Search modes - Boolean/Phrase	489
S27	S22 OR S23 OR S24 OR S25 OR S26	Expanders - Apply equivalent subjects Search modes - Boolean/Phrase	963 171
S26	(nurs* or anesthetist* or anesthesiologist* or ((nurs* or staff or personnel) N1 (anesthe* or anaesthe*)))	Expanders - Apply equivalent subjects Search modes - Boolean/Phrase	963 171
S25	(MH "Anesthesiologists")	Expanders - Apply equivalent subjects Search modes - Boolean/Phrase	1 634
S24	(MH "Anesthetists")	Expanders - Apply equivalent subjects Search modes - Boolean/Phrase	529
S23	"nurse anesthetists"	Expanders - Apply equivalent subjects Search modes - Boolean/Phrase	2 974
S22	(MH "Nurses")	Expanders - Apply equivalent subjects Search modes - Boolean/Phrase	68 437
S21	S15 OR S16 OR S17 OR S18 OR S19 OR S20	Expanders - Apply equivalent subjects Search modes - Boolean/Phrase	66 870
S20	((perioperative or peri-operative or per-operative or peri-surgical or perisurgical or intraoperative or intra-operative or peroperative or surg* or perianesthe*) N2 (care or nurs* or period*))	Expanders - Apply equivalent subjects Search modes - Boolean/Phrase	66 825
S19	(MH "Perianesthesia Nurses")	Expanders - Apply equivalent subjects Search modes - Boolean/Phrase	76
S18	(MH "Perianesthesia Nursing")	Expanders - Apply equivalent subjects Search modes - Boolean/Phrase	3 320
S17	(MH "Perioperative Nurses")	Expanders - Apply equivalent subjects	113

		Search modes - Boolean/Phrase	
S16	(MH "Perioperative Nursing") OR (MH "Operating Room Nursing")	Expanders - Apply equivalent subjects Search modes - Boolean/Phrase	14 728
S15	(MH "Perioperative Care")	Expanders - Apply equivalent subjects Search modes - Boolean/Phrase	12 382
S14	S8 OR S9 OR S10 OR S11 OR S12 OR S13	Expanders - Apply equivalent subjects Search modes - Boolean/Phrase	231 456
S13	(sedat* or anesthe* or anaesthe* or ((anesthe* or anaesthe*) N1 (opioid-free or "opioid free" or opiate-free or "opiate free")))	Expanders - Apply equivalent subjects Search modes - Boolean/Phrase	126 515
S12	(analges* or ((pain or analges*) N2 (management or relie*)))	Expanders - Apply equivalent subjects Search modes - Boolean/Phrase	111 075
S11	(MH "Anesthesia")	Expanders - Apply equivalent subjects Search modes - Boolean/Phrase	13 179
S10	(MH "Analgesics") OR (MH "Analgesics, Opioid+")	Expanders - Apply equivalent subjects Search modes - Boolean/Phrase	57 495
S9	(MH "Analgesia")	Expanders - Apply equivalent subjects Search modes - Boolean/Phrase	7 351
S8	(MH "Pain Management")	Expanders - Apply equivalent subjects Search modes - Boolean/Phrase	14 937
S7	S1 OR S2 OR S3 OR S4 OR S5 OR S6	Expanders - Apply equivalent subjects Search modes - Boolean/Phrase	329 481
S6	((buprenorphine* or methadone* or naltrexone* or opiate* or opioid*) N3 ("maintenan* therap*" or "maintenan* program*" or "maintenance treatment*" or "replacement therap*" or "substitution treatment*" or "substitution therap*"))	Search modes - Boolean/Phrase	2 591
S5	"opiate substitution treatment"	Search modes - Boolean/Phrase	65
S4	(addict* or ((opioid* or opiate* or morphine* or	Search modes - Boolean/Phrase	228 136

	heroin* or opium* or substance* or drug*) N2 (disorder* or misus* or use* or using or addict* or abuse* or dependen*)))		
S3	(MH "Substance Dependence")	Search modes - Boolean/Phrase	10 384
S2	(MH "Narcotics+")	Search modes - Boolean/Phrase	42 351
S1	(MH "Substance Use Disorders+")	Search modes - Boolean/Phrase	185 322
