# Supplementary material for: Intraoperative pain management for patients undergoing medication-assisted rehabilitation: a scoping review
Source: BMC Anesthesiol. 2025 Dec 10;26:40. doi: 10.1186/s12871-025-03538-5 (PMC12801853; doi:10.1186/s12871-025-03538-5)
Supplement: Supplementary file 2 — Supplementary Material 1: Appendix 2. Updated search 2025. [file 12871_2025_3538_MOESM2_ESM.pdf]

Database: Ovid MEDLINE(R) ALL <1946 to April 08, 2025>
Search Strategy:
1 exp Opioid-Related Disorders/ (37384)
2 Substance-Related Disorders/ (110482)
3 (addict* or ((opioid* or opiate* or morphine* or heroin* or opium* or substance* or drug*) adj2 (disorder* or misus* or use* or using or addict* or abuse* or dependen*))).ti,ab. (387045)
4 Opiate Substitution Treatment/ (5597)
5 ((buprenorphine* or methadone* or naltrexone* or opiate* or opioid*) adj3 ("maintenan* therap*" or "maintenan* program*" or "maintenance treatment*" or "replacement therap*" or "substitution treatment*" or "substitution therap*")).ti,ab. (5587)
6 1 or 2 or 3 or 4 or 5 (432266)
7 Pain Management/ (44699)
8 Analgesics, Opioid/ (66102)
9 exp Analgesics/ (614891)
10 (analges* or ((pain or analges*) adj2 (management or relie*))).ti,ab. (224059)
11 Anesthesia/ (68033)
12 (sedat* or anesthe* or anaesthe* or ((anesthe* or anaesthe*) adj1 (opioid-free or "opioid free" or opiate-free or "opiate free"))).ti,ab. (495083)
13 7 or 8 or 9 or 10 or 11 or 12 (1193212)
14 Perioperative Care/ (17198)
15 Perioperative Nursing/ (7074)
16 Intraoperative Care/ (18015)
17 ((perioperative or peri-operative or intraoperative or intra-operative or peroperative or surg* or perianesthe*) adj2 (care or nurs*)).ti,ab. (35315)
18 14 or 15 or 16 or 17 (72625)
19 6 and 13 and 18 (759)
20 limit 19 to (yr="2013 -Current" and (danish or english or norwegian or swedish)) (538)
21 Nurse Anesthetists/ (3048)
22 Nurses/ (48499)
23 Anesthetists/ (551)
24 Anesthesiologists/ (2313)
25 (nurs* or anesthetist* or anesthesiologist* or (nurs* adj1 an?esthe*)).ti,ab. (595351)
26 21 or 22 or 23 or 24 or 25 (609755)
27 6 and 13 and 18 and 26 (179)
28 limit 27 to (yr="2013 -Current" and (danish or english or norwegian or swedish)) (118)
29 limit 28 to dt=20231207-20250408 (13)

Database: Embase <1974 to 2025 April 08>
Search Strategy:
1 opiate addiction/ (33233)
2 drug dependence/ (80132)
3 (addict* or ((opioid* or opiate* or morphine* or heroin* or opium* or substance* or drug*) adj2 (disorder* or misus* or use* or using or addict* or abuse* or dependen*))).ti,ab. (538014)
4 opiate substitution treatment/ (3584)
5 ((buprenorphine* or methadone* or naltrexone* or opiate* or opioid*) adj3 ("maintenan* therap*" or "maintenan* program*" or "maintenance treatment*" or "replacement therap*" or "substitution treatment*" or "substitution therap*")).ti,ab. (7644)
6 1 or 2 or 3 or 4 or 5 (570546)
7 analgesia/ (171762)
8 analgesic agent/ (111378)
9 anesthesia/ (121680)
10 (analges* or ((pain or analges*) adj2 (management or relie*))).ti,ab. (319908)
11 (sedat* or anesthe* or anaesthe* or ((anesthe* or anaesthe*) adj1 (opioid-free or "opioid free" or opiate-free or "opiate free"))).ti,ab. (660098)
12 7 or 8 or 9 or 10 or 11 (1018866)
13 perioperative care/ or peroperative care/ (20651)
14 perioperative period/ or intraoperative period/ (124585)
15 perioperative nursing/ or operating room nursing/ or perianesthesia nursing/ (6441)
16 peroperative care/ (15928)
17 ((perioperative or peri-operative or per-operative or peri-surgical or perisurgical or intraoperative or intra-operative or peroperative or surg* or perianesthe*) adj2 (care or nurs* or period*)).ti,ab. (81462)
18 13 or 14 or 15 or 16 or 17 (208056)
19 6 and 12 and 18 (2450)
20 limit 19 to ((danish or english or norwegian or swedish) and yr="2018 -Current") (1460)
21 nurse anesthetist/ (2890)
22 Nurses/ (165097)
23 anesthesist/ (28502)
24 anesthesiologist/ (14947)
25 (nurs* or anesthetist* or anesthesiologist* or ((nurs* or staff or personnel) adj1 an?esthe*)).ti,ab. (701550)
26 21 or 22 or 23 or 24 or 25 (753214)
27 6 and 12 and 18 and 26 (532)
28 limit 27 to ((danish or english or norwegian or swedish) and yr="2013 -Current") (386)
29 limit 28 to dc=20231207-20250408 (65)

EBSCOhost Tue, april 8, 2025 03:34:44 p.m.

#	Query	Limiters/Expanders	Last Run Via	Results
S33	S31 AND S32	Expanders - Apply equivalent subjects<br>Search modes - Proximity	Interface - EBSCOhost Research Databases<br>Search Screen - Advanced Search<br>Database - CINAHL	24
S32	EM 20231207-20250408	Limiters - Publication Date: 20130101-20251231; Language: Danish, English, Norwegian, Swedish<br>Expanders - Apply equivalent subjects<br>Search modes - Proximity	Interface - EBSCOhost Research Databases<br>Search Screen - Advanced Search<br>Database - CINAHL	289,864
S31	S7 AND S14 AND S21 AND S27	Limiters - Publication Date: 20130101-20251231; Language: Danish, English, Norwegian, Swedish<br>Expanders - Apply equivalent subjects<br>Search modes - Proximity	Interface - EBSCOhost Research Databases<br>Search Screen - Advanced Search<br>Database - CINAHL	266
S30	S7 AND S14 AND S21 AND S27	Expanders - Apply equivalent subjects<br>Search modes - Proximity	Interface - EBSCOhost Research Databases<br>Search Screen - Advanced Search<br>Database - CINAHL	504
S29	S7 AND S14 AND S21 AND S27	Limiters - Publication Date: 20130101-20231231; Language: Danish, English, Norwegian, Swedish<br>Search modes - Proximity	Interface - EBSCOhost Research Databases<br>Search Screen - Advanced Search<br>Database - CINAHL	243
S28	S7 AND S14 AND S21 AND S27	Expanders - Apply equivalent subjects<br>Search modes - Proximity	Interface - EBSCOhost Research Databases<br>Search Screen - Advanced Search<br>Database - CINAHL	Display

S27	S22 OR S23 OR S24 OR S25 OR S26	Expanders - Apply equivalent subjects<br>Search modes - Proximity	Interface - EBSCOhost Research Databases<br>Search Screen - Advanced Search<br>Database - CINAHL	Display
S26	(nurs* or anesthetist* or anesthesiologist* or ((nurs* or staff or personnel) N1 (anesthe* or anaesthe*)))	Expanders - Apply equivalent subjects<br>Search modes - Proximity	Interface - EBSCOhost Research Databases<br>Search Screen - Advanced Search<br>Database - CINAHL	Display
S25	(MH "Anesthesiologists")	Expanders - Apply equivalent subjects<br>Search modes - Proximity	Interface - EBSCOhost Research Databases<br>Search Screen - Advanced Search<br>Database - CINAHL	Display
S24	(MH "Anesthetists")	Expanders - Apply equivalent subjects<br>Search modes - Proximity	Interface - EBSCOhost Research Databases<br>Search Screen - Advanced Search<br>Database - CINAHL	Display
S23	"nurse anesthetists"	Expanders - Apply equivalent subjects<br>Search modes - Proximity	Interface - EBSCOhost Research Databases<br>Search Screen - Advanced Search<br>Database - CINAHL	Display
S22	(MH "Nurses")	Expanders - Apply equivalent subjects<br>Search modes - Proximity	Interface - EBSCOhost Research Databases<br>Search Screen - Advanced Search<br>Database - CINAHL	Display
S21	S15 OR S16 OR S17 OR S18 OR S19 OR S20	Expanders - Apply equivalent subjects<br>Search modes - Proximity	Interface - EBSCOhost Research Databases<br>Search Screen - Advanced Search<br>Database - CINAHL	Display
S20	((perioperative or peri-operative or per-operative or peri-surgical or perisurgical or intraoperative or intra-operative or peroperative or surg* or perianesthe*)	Expanders - Apply equivalent subjects<br>Search modes - Proximity	Interface - EBSCOhost Research Databases<br>Search Screen - Advanced Search<br>Database - CINAHL	Display

	N2 (care or nurs* or period*))			
S19	(MH "Perianesthesia Nurses")	Expanders - Apply equivalent subjects<br>Search modes - Proximity	Interface - EBSCOhost Research Databases<br>Search Screen - Advanced Search<br>Database - CINAHL	Display
S18	(MH "Perianesthesia Nursing")	Expanders - Apply equivalent subjects<br>Search modes - Proximity	Interface - EBSCOhost Research Databases<br>Search Screen - Advanced Search<br>Database - CINAHL	Display
S17	(MH "Perioperative Nurses")	Expanders - Apply equivalent subjects<br>Search modes - Proximity	Interface - EBSCOhost Research Databases<br>Search Screen - Advanced Search<br>Database - CINAHL	Display
S16	(MH "Perioperative Nursing") OR (MH "Operating Room Nursing")	Expanders - Apply equivalent subjects<br>Search modes - Proximity	Interface - EBSCOhost Research Databases<br>Search Screen - Advanced Search<br>Database - CINAHL	Display
S15	(MH "Perioperative Care")	Expanders - Apply equivalent subjects<br>Search modes - Proximity	Interface - EBSCOhost Research Databases<br>Search Screen - Advanced Search<br>Database - CINAHL	Display
S14	S8 OR S9 OR S10 OR S11 OR S12 OR S13	Expanders - Apply equivalent subjects<br>Search modes - Proximity	Interface - EBSCOhost Research Databases<br>Search Screen - Advanced Search<br>Database - CINAHL	Display
S13	(sedat* or anesthe* or anaesthe* or ((anesthe* or anaesthe*) N1 (opioid-free or "opioid free" or opiate-free or "opiate free")))	Expanders - Apply equivalent subjects<br>Search modes - Proximity	Interface - EBSCOhost Research Databases<br>Search Screen - Advanced Search<br>Database - CINAHL	Display
S12	(analges* or ((pain or analges*) N2 (management or relie*)))	Expanders - Apply equivalent subjects<br>Search modes - Proximity	Interface - EBSCOhost Research Databases<br>Search Screen - Advanced Search<br>Database - CINAHL	Display

S11	(MH "Anesthesia")	Expanders - Apply equivalent subjects<br>Search modes - Proximity	Interface - EBSCOhost Research Databases<br>Search Screen - Advanced Search<br>Database - CINAHL	Display
S10	(MH "Analgesics") OR (MH "Analgesics, Opioid+")	Expanders - Apply equivalent subjects<br>Search modes - Proximity	Interface - EBSCOhost Research Databases<br>Search Screen - Advanced Search<br>Database - CINAHL	Display
S9	(MH "Analgesia")	Expanders - Apply equivalent subjects<br>Search modes - Proximity	Interface - EBSCOhost Research Databases<br>Search Screen - Advanced Search<br>Database - CINAHL	Display
S8	(MH "Pain Management")	Expanders - Apply equivalent subjects<br>Search modes - Proximity	Interface - EBSCOhost Research Databases<br>Search Screen - Advanced Search<br>Database - CINAHL	Display
S7	S1 OR S2 OR S3 OR S4 OR S5 OR S6	Expanders - Apply equivalent subjects<br>Search modes - Proximity	Interface - EBSCOhost Research Databases<br>Search Screen - Advanced Search<br>Database - CINAHL	Display
S6	((buprenorphine* or methadone* or naltrexone* or opiate* or opioid*) N3 ("maintenan* therap*" or "maintenan* program*" or "maintenance treatment*" or "replacement therap*" or "substitution treatment*" or "substitution therap*"))	Search modes - Proximity	Interface - EBSCOhost Research Databases<br>Search Screen - Advanced Search<br>Database - CINAHL	Display
S5	"opiate substitution treatment"	Search modes - Proximity	Interface - EBSCOhost Research Databases<br>Search Screen - Advanced Search<br>Database - CINAHL	Display
S4	(addict* or ((opioid* or opiate* or morphine* or	Search modes - Proximity	Interface - EBSCOhost Research Databases	Display

	heroin* or opium* or substance* or drug*) N2 (disorder* or misus* or use* or using or addict* or abuse* or dependen*)))		Search Screen - Advanced Search<br>Database - CINAHL	
S3	(MH "Substance Dependence")	Search modes - Proximity	Interface - EBSCOhost Research Databases<br>Search Screen - Advanced Search<br>Database - CINAHL	Display
S2	(MH "Narcotics+")	Search modes - Proximity	Interface - EBSCOhost Research Databases<br>Search Screen - Advanced Search<br>Database - CINAHL	Display
S1	(MH "Substance Use Disorders+")	Search modes - Proximity	Interface - EBSCOhost Research Databases<br>Search Screen - Advanced Search<br>Database - CINAHL	Display
